# Parasites and diet as main drivers of the Malagasy gut microbiome richness and function

**DOI:** 10.1038/s41598-021-96967-4

**Published:** 2021-09-03

**Authors:** Stanislas Mondot, Philippe Poirier, Ahmed Abou-Bacar, Valentin Greigert, Julie Brunet, Céline Nourrisson, Milijaona Randrianarivelojosia, Jean-Louis Razafindrakoto, Eugene Morel, Rivo S. Rakotomalala, Marion Leclerc, Karine Le Roux, Céline Monot, Patricia Lepage, Ermanno Candolfi

**Affiliations:** 1 Université Paris-Saclay, INRAE, AgroParisTech, Micalis Institute, Domaine de Vilvert, 78350 Jouy-en-Josas, France; 2 Laboratoire de Parasitologie-Mycologie, CHU Clermont-Ferrand, 63000 Clermont-Ferrand, France; 3 Université Clermont Auvergne/Inserm U1071; USC-INRAE 2018, Microbes, Intestin, Inflammation et Susceptibilité de l’Hôte (M2iSH), 3iHP, Clermont-Ferrand, France; 4 Institut de Parasitologie et Pathologie Tropicale, Université de Strasbourg, Strasbourg, France; 5 Laboratoire d’Analyse Médicale, CHU PZaGa, Université de Mahajanga, Mahajanga, Madagascar; 6 Facultés des Sciences, Toliara, Madagascar; 7 Institut Pasteur de Madagascar, Antananarivo, Madagascar; 8 Service d’Hépato-Gastro-Entérologie, CHU Mahavoky Atsimo, Mahajanga, Madagascar

**Keywords:** Microbial ecology, Microbiome

## Abstract

Interactions between the prokaryotic microbiome and eukaryotic parasites in the vertebrate gut may affect overall host health and disease. While intertropical areas exhibit a high rate of parasites carriers, such interactions are understudied in these populations. Our objectives were to (1) describe the gut microbiome of individuals living in Madagascar, (2) identify potential associations between bacterial taxa and parasites colonizing the digestive tract and (3) highlight main determinants of the gut microbiota composition in this developing country. Metadata (socioeconomic, diet, clinical) and fecal samples were collected from 219 volunteers from North-West Madagascar (Mahajanga). Fecal microbiome was assessed through 16S rRNA gene sequencing and metabolomics, and related to dietary habits and parasites carriage. We highlight important Malagasy gut microbiome peculiarities. Out of three detected enterotypes, only one is similar to that observed in Westernized countries (*Ruminococcus*-driven). Functions associated with the two others (*Clostridium *sensu stricto-driven and *Escherichia/Shigella*-driven) are mostly directed toward amino acids biosynthesis and degradation, respectively. Diet and protozoan carriage were the main drivers of microbiota composition. High protozoan carriage was associated with higher diversity, richness and microbial functionalities. The gut microbiome of Malagasy strongly differs from that of Westernized countries. Asymptomatic protozoan carriage and dietary habits are the external factors with the deepest impact on gut microbiome. Further studies are needed to understand whether gut microbial richness constitute a predilection niche for protozoans colonization, due to their gazing features, or whether the parasites themselves induce a higher bacterial richness.

## Introduction

The trillions of bacteria colonizing our intestines are known to play key roles in human health, ranging from digestion, to immunity development, yet, complexity of the human gut microbiome is far from being fully decrypted. Most studies are performed in westernized countries and, when focusing on lower income countries, often aim at linking the gut microbiome with nutritional status. Hence, one of the first studied populations from rural Africa highlighted a specific microbiome in 14 children from Burkina Faso, geared toward degradation of polysaccharides^1^. Another study emphasized starch degradation capabilities of the gut microbiome in a Malawi children cohort (n = 83 infants)^2^. When analyzing the microbiota of adult population, isolated territories or people are often selected to describe the reciprocal impact of genetic versus environment on the gut ecosystem. Most of these studies describe a higher bacterial diversity and richness in these populations, either originating from the East African tropical savanna (n = 27 Hadza hunters-gathers^3^) or the southwestern Pacific Ocean (n = 40 individuals originating from two traditional societies of Papua New Guinea^4^).

Parasitic eukaryotes members of the human microbiome are often underestimated and underexplored, even though their importance in the gut ecosystem, mainly as immunity drivers, is widely accepted. Protozoans’ colonization ranges from 5 to 100% of the population, depending on geographical origin, with many carriers remaining asymptomatic. Some parasitic protozoa, such as *Entamoeba histolytica*, *Giardia intestinalis*, and *Tritrichomonas suis*, can modify intestinal mucus abundance and composition, enabling them to penetrate the mucus barrier during pathogenesis^5^. Many gut protozoa can also damage the epithelium during pathogenesis (disruption of tight junctions, cell invasion and destruction)^6^. Both mucosal and epithelial damages will directly alter the host's interaction with their microbiome during infection. However, asymptomatic parasitic colonization also impacts the host microbiome and one of the most studied organisms is *Blastocystis* sp. (abbreviated as *Blastocystis* in the present paper). While historically considered as parasite and assumed to have a detrimental effect on the host organism, the vision of a potential commensalism and beneficial role of *Blastocystis* starts to emerge^7^. A recurrent finding is the robust link between gut colonization by *Blastocystis* and higher microbiome diversity and/or richness^8–12^, together with a negative association with the *Bacteroides* genus abundance and *Bacteroides* enterotypes^9,12,13^.

Still, these results are seen through the prism of westernized countries (France, Sweden, Belgium and Mexico) and compiled data from shotgun metagenomic studies. Considering the higher rate of parasitic carriers in intertropical area, we focused our research on volunteers from Madagascar and previously described a wide panel of intestinal parasites^14^. Protozoa were the most prevalent with 72.8% of the population colonized whereas helminths and microsporidia were detected in less than 10% of the volunteers. *Blastocystis* was the most prevalent protozoa followed by various amoebas and flagellates.

The objectives of the present study were to (1) describe the gut microbiota composition of individuals living in Madagascar, (2) identify potential associations between gut bacterial taxa and parasites colonizing the digestive tract and (3) highlight main determinants of the gut microbiota composition in this developing country.

It is, to our knowledge the first paper intending to describe the general gut microbiome profiles of healthy adults from Madagascar, and, together with the Flemish gut project^12^, linking parasitic status to microbiome in a cohort of more than 200 individuals.

## Results

### Malagasy population exhibits a specific gut microbiome

A total of 6428 OTUs was detected in the fecal samples provided by 219 Malagasy individuals. On average, a Malagasy microbiome harboured 439 ± 106 OTUs classified in 12 microbial phyla. Main phyla were affiliated to *Firmicutes* (58.24% ± 19.96), *Bacteroidetes* (15.90% ± 13.65), *Proteobacteria* (14.01% ± 19.80), *Actinobacteria* (5.62% ± 6.07) and *Spirochaetes* (1.48% ± 4.61). Microbial diversity and richness indices (Simpson: 0.90 ± 0.10 and number of observed OTUs: 439 ± 106, respectively) were high. The bacterial load estimated by qPCR depicted a dense bacterial ecosystem composed on average of 1.67 × 10^10^ CFU/g of feces (1.14 × 10^6^–2.05 × 10^12^ CFU/g).

**Figure 1:**
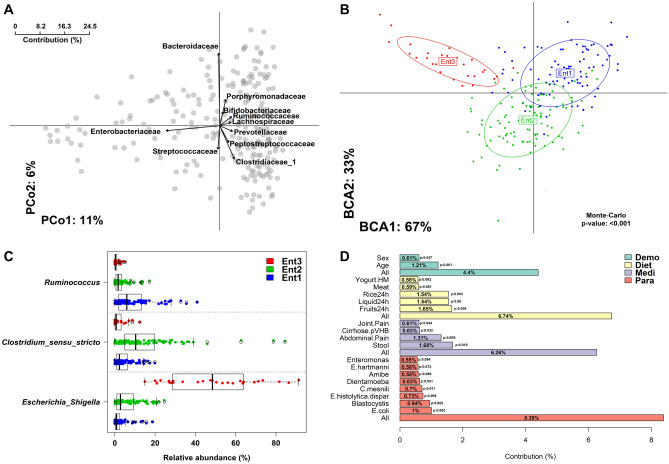
Malagasy gut microbiota general description and enterotypes classification. (**A**) Bacterial families (n = 10) contributing the most to the dissimilarity observed within the 219 microbiomes. Taxa contribution was determined using canonical correspondence analysis of family abundances and was plotted on the two first PCoA dimensions. Arrows length is proportional to family contribution (%). Dots indicate individual’s location on PCoA. (**B**) PCA inter-class analysis depicting the enterotype stratification of individuals from Madagascar. Each dot represents a fecal sample. (**C**) Bacterial genera driving the most the enterotype description. Bacterial genera were selected following a random forest classification based on enterotypes stratification. (**D**) Factors associated with the inter-individual variation based on the gut microbiota composition. A total of 19 factors (FDR < 0.1) including medical, demographical, dietary and protozoan carriage were associated with inter-individual variation of the gut microbiota composition. The bar plot indicates the contribution (%) of each factor at explaining the inter-individual variation observed within the faecal bacterial composition of Malagasy individuals.

Ten bacterial families were identified as taxa contributing the most to the variance observed between microbiota profiles (96% of the β-diversity variance explained) (Fig. 1A) and were affiliated to *Enterobacteriaceae*, *Peptostreptococcaceae*, *Clostridiaceae* 1, *Porphyromonadaceae*, *Streptococcaceae*, *Ruminococcaceae*, *Bacteroidaceae*, *Prevotellaceae*, *Bifidobacteriaceae*, and *Lachnospiraceae*. Three distinct clusters or enterotypes (Ent) were statistically detected within the microbiome of the Malagasy population (Fig. 1B). Enterotypes stratification was mainly supported by the relative abundance of *Ruminococcus* (Ent1—n = 92 individuals), *Clostridium *sensu stricto (Ent2—n = 100 individuals) and *Escherichia*/*Shigella* (Ent3—n = 27 individuals) genera (Fig. 1C). Ent1 was driven by *Ruminococcus bromii* (100% identity). An uncultured rumen clone (98%—EF436397) and *Clostridium disporicum* (100%) were associated with Ent2. *Escherichia coli* (100%) was found more abundant in Ent3. Both bacterial diversity and richness were significantly lower in Ent3 as compared to Ent1 and Ent2 (p = 1e^−13^ and 1.1e^−09^ and p = 8e^−07^ and 8e^−06^ respectively) (Additional file 1: Fig. S1A). However, the bacterial load estimated by qPCR was not statistically different between the three enterotypes (ANOVA p = 0.19). (Additional file 1: Fig. S1B).

### Intestinal colonization by parasites strongly affects the gut microbiome

The contribution of demographical (n = 5), medical (n = 8), dietary (n = 7) and parasitism status (n = 14) factors on inter-individual microbial composition (β-diversity) was assessed by PERMANOVA analysis. At a false discovery rate of 0.1, 19 factors were associated with dissimilarity in microbial composition (Fig. 1D). Altogether, these factors explained 25.79% of the observed variation between individuals. Neither bacterial diversity nor bacterial richness correlated with age, gender or BMI, while variation in the microbiota composition within Malagasy population was slightly but significantly affected by individual’s age (1% of total microbiota variance).

**Figure 2:**
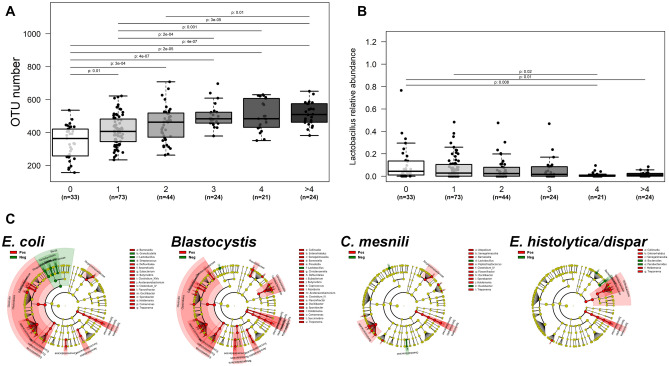
Association between intestinal colonization by parasites and the gut microbiome richness and composition. (**A**) Microbial richness (OTU number) as function of cumulative number of different protozoans detected in an individual (ranged from 0 to > 4). (**B**) Relative abundance of Lactobacillus genus as function of the cumulative number of protozoans detected in an individual (ranged from 0 to > 4). (**C**) Cladogram representation of bacterial groups significantly increased (in red) or decreased (in green) in the presence of the four main parasitic colonizers (*Entamoeba coli*, *Blastocystis* sp., *Chilomastix mesnili* and *Entamoeba histolytica*/*dispar*).

Parasitic status accounted for most of the bacterial microbiome dissimilarity (8.39%) and especially *Entamoeba coli* (1%), *Blastocystis* (0.94%), *Entamoeba histolytica*/*dispar* (0.73%) and *Chilomastix mesnili* (0.7%). Within our cohort, 85% of individuals were colonized by one or more parasites. *Blastocystis* was the most prevalent (77.6% of the cohort) and frequently associated with *Dientamoeba fragilis* (22.8%). While most parasites were associated with higher bacterial richness, microbiome diversity was also significantly increased in individuals colonized by *Blastocystis* sp., *Entamoeba coli*, and *Enteromonas* (Additional file 2: Fig. S2A). On the other hand, individuals positive for *Blastocystis*, *Ameoba* and *C*. *mesnili* displayed lower bacterial load. Strikingly, cumulative parasite colonization of the Malagasy gut increased bacterial richness and diversity (Fig. 2A). Relative abundances of 21 taxa shifted according to the presence of parasites with *Blastocystis* and *E*. *coli* having the greatest impact on gut microbiome. Colonized patients had higher relative abundance of commensal bacteria but lower proportions of *Streptococcus* and *Lactobacillus* (Fig. 2C). A higher parasitic cumulative diversity was associated with a significantly lower abundance of *Lactobacillus* (Fig. 2B), and with a low sanitary level (Additional file 2: Fig. S2B).

The proportion of individuals positive for *Blastocystis*, *D*. *fragilis*, *E*. *nana* and *E*. *coli* was lower in the *Escherichia*/*Shigella* Ent3 as compared to others (Additional file 2: Fig. S2C). On the contrary, most of the individuals colonized by three or more different parasites clustered within the *Ruminococcus*-driven Ent1 (Additional file 2: Fig. S2D).

We further investigated the relative importance of parasites symptomatology and carriage on the gut bacterial composition. While for most parasites, asymptomatic and symptomatic carriers share a similar microbiome, individuals colonized with *Blastocystis* and presenting gut symptoms of blastocystosis exhibited lower relative abundance of both *Acetanaerobacterium* and *Flavonifractor* (Additional file 2: Fig. S2E) than asymptomatic carriers.

### Dietary habits strongly impact the Malagasy gut microbiome

**Figure 3:**
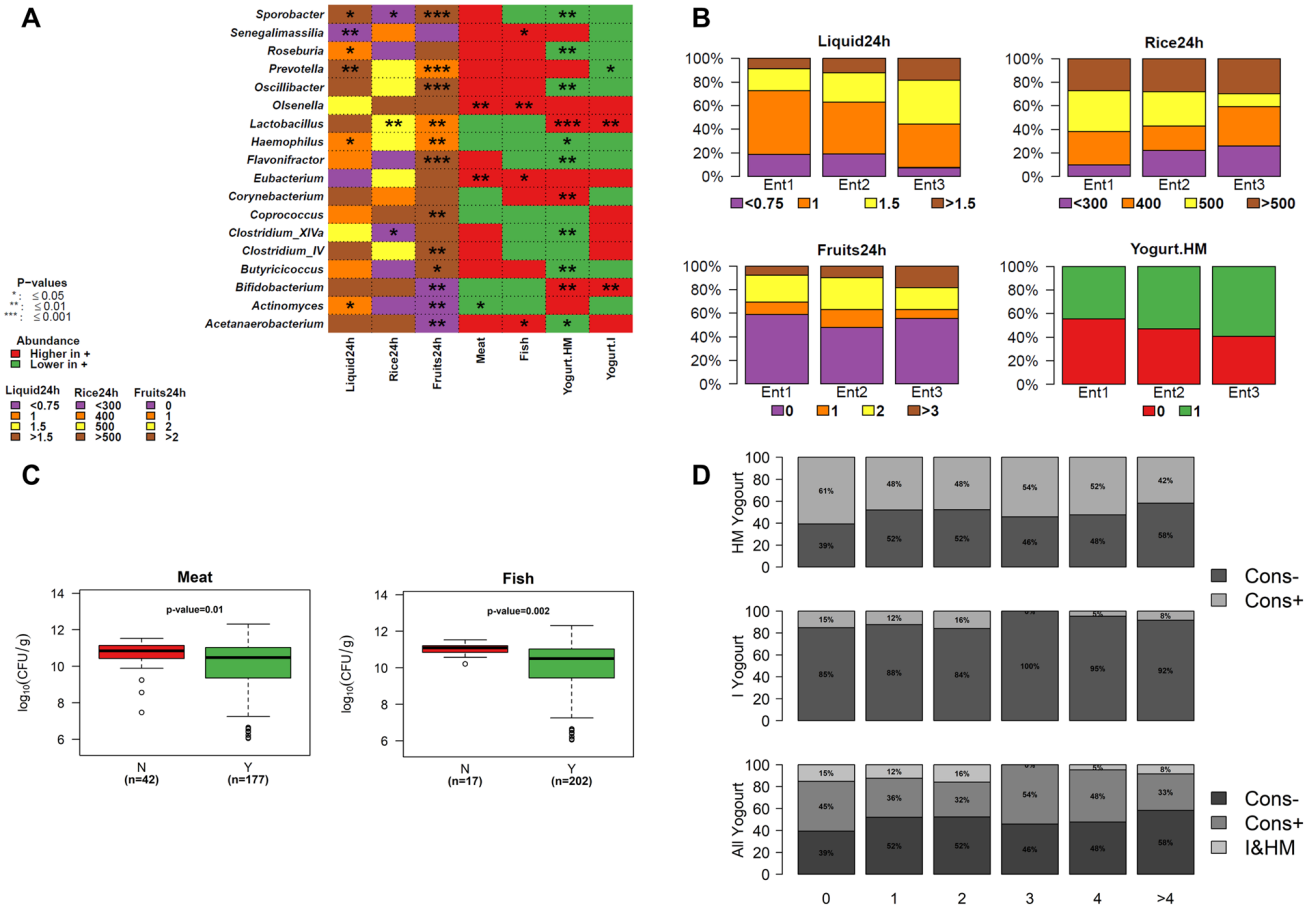
Impact of dietary habits on gut microbiome composition in Malagasy volunteers. (**A**) Heatmap depicting the bacterial genera having an abundance modulated by daily dietary habits. Liquid consumption is expressed in litre (L), rice in gram (g), fruits in number and other dietary information as yes/no question. Red, blue and green boxes indicate whether the abundance is higher, equal or lower in individuals positive for the listed food type. ANOVA (group > 2) and Wilcoxon test (group = 2) were run all over genera count dataset according to diet. Only boxes tagged with a star are considered statistically informative. The heatmap was generated using R software (https://cran.r-project.org/) with in-house code that uses basic functions included in "stats" and "graphics" packages. It uses genera abundance (as listed in rows) and highlights, for daily consumption of liquid, rice and fruit items, the category that are the most significantly different from others. For Meat, Fish, Yogurt-HM and Yogurt-I items, the heatmap indicates genera whose abundances are increased/decreased in individuals consuming these items. (**B**) Proportion of individuals included in each enterotype according to food type’s consumption. (**C**) Bacterial load distribution among meat and fish eaters (Y) as compared to non-eaters (N). (**D**) Proportion of individuals eating either yogurt (Cons+) (home-made yogurt, industrial yogurt or both (I&HM)), as function of cumulative number protozoans detected in an individual.

Dietary habits had a significant impact (6.74%) on the inter-individual microbiota composition (Fig. 1D). From all collected dietary information, daily liquid, rice and fruit consumptions had the most significant influence on gut microbiota variance (1.54%, 1.54% and 1.64% respectively). Low liquid consumption (< 1 L/day) was associated with significantly higher proportions of *Senegalimassilia*, *Roseburia*, *Haemophilus* and *Actinomyces* (Fig. 3A). Microbiota of individuals not eating fruits harboured higher percentages of bacteria from *Actinomyces*, *Bifidobacterium* and *Acetanaerobacterium* (Fig. 3A) whereas moderate fruit eaters (1–2) displayed a higher abundance of *Prevotella*, *Haemophilus* and *Lactobacillus*. Daily consumption of 3 or more fruits increased the abundance of *Sporobacter*, *Oscillibacter*, *Flavonifactor*, *Coprococcus*, *Clostridium IV* and *Butyricicoccus* genera.

Malagasy gut bacterial load was significantly lower in individuals eating either meat or fish meals (Fig. 3C). Individuals eating both meat and fish harboured higher proportions of *Eubacterium* and *Olsenella* than non-eaters. Microbiota of meat eaters was characterized by less *Actinomyces*, whereas fish eaters’ microbiota exhibited more *Acetanaerobacterium* and *Senegalimassilia*. Low to moderate daily rice consumers (< 400 g/day) had higher relative abundance of bacterial species related to *Lactobacillus*, *Clostridium XIVa* and *Sporobacter* (Fig. 3A).

Yogurt consumption had also a deep impact on the microbiota. Both homemade and industrialized yogurt consumption led to significantly higher abundance of *Bifidobacterium* and *Lactobacillus*. Thirteen other genera were significantly associated with homemade yogurt consumption, most of them being less represented. Dietary habits were overall similar between Ent1 and Ent2 individuals whereas Ent3 was characterized by higher volume drinkers, lesser fruit and rice eaters and more homemade yogurt consumers (Fig. 3B).

A high proportion of parasites-free volunteers consumed homemade yogurt (61%), while highly parasites-colonized individuals did not (58%) (Fig. 3D).

### Metabolism of the gut microbiome in Malagasy individuals

**Figure 4:**
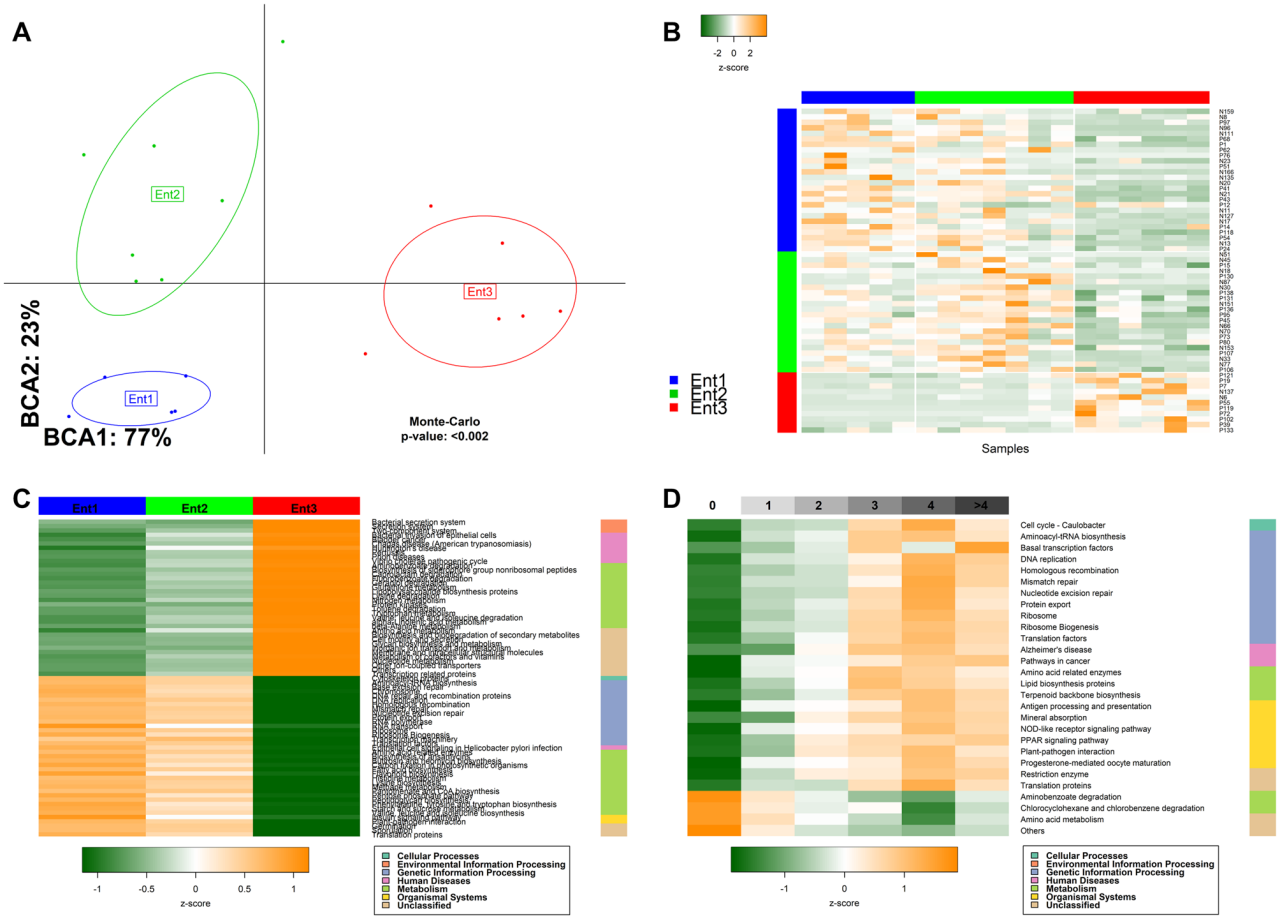
Metabolomic specificities of the microbiome in Malagasy volunteers. Eighteen volunteers were selected according to their enterotypes affiliation (nEnt1 = 5, nEnt2 = 7, nEnt3 = 6) and analysed through metabolomic approach. (**A**) The inter-class analysis computed upon PCA data emphasizes the stratification of these metabolomes in three clusters, similarly to microbiome enterotypes distribution. (**B**) Metabolic peaks (n = 51) discriminating the Malagasy metabolomes according to enterotypes stratification. (**C**) Metabolic functions inferred from PICRUSt that pointed out the functional specificities associated with each enterotypes. Functions listed in the heatmap had a significantly different distribution between enterotypes (p ≤ 0.01) and were selected according to Kruskal Walis’s test followed by a post-hoc Dunn’s test assessing the intergroup (enterotypes) comparison. All tests were corrected for false discovery rate. (**D**) Functions differentially distributed among protozoan carrier types (0 to > 4).

A global metabolomic approach has been applied on a subset of fecal samples (nEnt1 = 5, nEnt2 = 7, nEnt3 = 6) and 256 peaks were detected. Similarly to gut microbiota composition, metabolomic profiles allowed distinguishing three groups of individuals (Fig. 4A). A total of 59 peaks, belonging to six classes (Amino Acids, Peptides, and Analogues—Aromatic Compounds—Aliphatic Compounds—Carboxylic acids and derivatives—Lipids—Organic Acids and Derivatives) were differentially represented between enterotypes (Fig. 4B). Specific metabolites associated with each enterotype are described in Additional file 4 (Table S2). Metabolites relating to (1) phenylalanine, tyrosine and tryptophan biosynthesis, (2) tryptophan metabolism, (3) valine, leucine, isoleucine biosynthesis and (4) lysine biosynthesis were specific of *Clostridium*-driven Enterotype (Ent2). On the other hand, Pantothenate and CoA biosynthesis as well as beta-Alanine metabolism were distributed in both Ent2 and *Escherichia*-driven Ent3. Finally, metabolites associated with *Ruminococcus*-driven Ent1 related to metabolism more than biosynthesis (Glycine, serine, threonine metabolism—Cyanoaminoacid metabolism—Riboflavin metabolism).

A statistical inference of metabolic functions with 16S rRNA data was also performed with PICRUSt to cover the overall population. The most important shift in terms of microbiome gut functions was observed between Ent3 and the two other Enterotypes (Fig. 4C and Additional files 4 and 6: Tables S2 and S4). Xenobiotics biodegradation and metabolism such as aminobenzoate, fluorobenzoate, toluene degradation were enriched in Ent3. The metabolism of Terpenoids and Polyketides in Ent3 was marked by the biosynthesis of siderophore group non-ribosomal peptides whereas Ent1 and Ent2 harboured more functions classified in the biosynthesis of ansamycins, butirosin and neomycin. Ent3 was characterized by a lower abundance of functions related to pentose phosphate pathway, starch and sucrose metabolism. The amino acid metabolism was totally directed to the catabolism of gluthatione, lysine, tryptophan, valine, leucine and isoleucine. On the opposite, Ent1 and 2 were more prone to the biosynthesis of these amino acid units. The increased amino acid biosynthesis observed in Ent1 and 2 was interconnected with enriched protein export capabilities. Functions related to replication and repair, transcription and translation were less represented in Ent3. Similar results were observed for membrane transport and signal transduction functions.

Metabolic functions deduced from PICRUSt were also screened in Malagasy gut microbiomes in accordance to increasing asymptomatic protozoans carriage (Fig. 4D and Additional files 5 and 7: Tables S3 and S5). Microbial functions such as replication and repair, translation, amino acid and lipid metabolism, protein export and terpenoid backbone biosynthesis were enriched in individuals with higher protozoans carriage (n ≥ 3). On the opposite, xenobiotics biodegradation and metabolism such as aminobenzoate and chlorocyclohexane and chlorobenzene degradation together with unclassified amino acid metabolism related functions were increased within microbiome of low protozoans carriers (n < 3).

## Discussion

The gut microbiome of people from the tropical region of Mahajanga, an urban community of Madagascar, strongly differs from that of westernized countries or isolated populations. While westernized countries microbiota can be clustered into three or four enterotypes^15^ driven by *Bacteroides*, *Prevotella* and *Ruminococcus*, only the latter genus could be described as an enterotype driver in this population. However, the microbiome was still statistically stratified into three enterotypes, the other two being driven by *Escherichia*/*Shigella* and *Clostridium *sensu stricto. Yet while *Prevotella* still accounts for approximately 7% in average of total bacteria in the overall population (median = 3%), proportions of *Bacteroides* are lower than 5% (4.4% and median = 0.99%).

Similarly, Pasolli et al. applied shotgun metagenomic gut microbiome profiling of two rural communities with non-Western lifestyles from north-eastern Madagascar and detected that the species-level genome bins profiles of the Madagascar population were profoundly different from that of Western-style populations^16^. A substantial proportion of genomic species related to *Prevotella* while *Bacteroides* was nearly absent as compared to westernized countries. Both our study and Pasolli et al. highlighted a depletion of *Alistipes*, *Parabacteroides* and *Akkermansia* in the Malagasy gut microbiomes.

Diet strongly explains this distribution. High proteins and animal fat intake has previously been associated with higher abundance of the *Bacteroides* dominant enterotype^17^. Its absence in Madagascar probably reflects a diet mostly based on rice. Fruits consumption had the highest impact on microbiome variance within the population. Fruits and vegetables are important sources of dietary fibers that will undergo microbial metabolism in the digestive tract. Fibers are known to increase microbiota diversity^18,19^. Consuming two or more fruits per day in the present population was associated with higher levels of *Firmicutes* and butyrate-producing bacteria such as *Prevotella* or *Coprococcus*. The gut microbiota of individuals ingesting important rice portions (≥ 500 g/day) was enriched in *Ruminococcus*, *Sporobacter* and rice consumption was higher in individuals belonging to Ent1. Individuals within this enterotype also ingested less yogurt (both home-made and commercial). Yogurt was associated with significantly higher proportions of *Lactobacillus* and *Bifidobacterium*, and lower levels of *Prevotella* and *Enterorhabdus*.

At a functional level, Ent1 and Ent2 were mostly enriched in functions dedicated to the biosynthesis of several amino acids as well as central bacterial cell mechanisms such as replication and repair, translation and transcription. On the opposite, numerous functions sustaining degradation of amino acid units and xenobiotics metabolism were enriched in Ent3 as compared to Ent1 and Ent2. This observation is coupled with higher metabolism of nitrogen compounds. The energy metabolism was more directed towards nitrogen metabolism in Ent3 while carbon fixation in photosynthetic organisms and methane metabolism were increased in Ent1 and 2. Previous reports have indicated that aminobenzoate metabolism influences the microbial composition and intermediaries of aminobenzoate degradation are able to enhance a stress response^20^ and promote the growth and virulence of *Enterobacteriaceae*^21^. Similarly, the metabolism of aromatic amino acid such as tryptophan, over-represented in Ent3, has been documented for the noxious potential of derivative compounds issued from tryptophan degradation on the immune system and neuronal functions^22,23^.

Ent3 harboured less functions classified in the biosynthesis of ansamycins, butirosin and neomycin. Neomycin is a well-characterized antibiotic with excellent activity against Gram-negative bacteria. This could partly explain the *Escherichia/Shigella* specificity of Ent3.

A main finding of our study is the robust link between the cumulative number of colonizing parasites and the increase gut microbial diversity and richness. An association between *Blastocystis* colonization and microbiota diversity has previously been described in both westernized and developing countries^12,13^. In more than 600 healthy European (Flemish adults cohort), Tito et al. reported a higher bacterial diversity associated with the detection of *Blastocystis*. Moreover, *Blastocystis* prevalence was unevenly distributed over Westernized country-type enterotypes, with the *Bacteroides* clusters comprising significantly less *Blastocystis*-carriers compared with both *Ruminococcaceae* and *Prevotella* enterotypes. This is in line with our observation since > 75% of our cohort was colonized by *Blastocystis* and we would not depict a *Bacteroides* cluster in this population, underlying the link between this enterotype and the absence of *Blastocystis*. We here highlight for the first time that gut colonization by a multi-species community of parasites is associated with higher microbiota richness, increasing with the number of different parasites (from 1 to more than 5 different parasites species). Interestingly, while microbial richness increased with the number of parasites colonizing the gut, this was inversely associated with proportions of *Lactobacillus*, which were significantly increased in yogurt consumers. Hence, one could speculate that yogurt consumption might, in this specific population, be disadvantageous for parasitic colonization and microbiome diversity and richness. An impact of *Lactobacillus* on fungi and parasites growth was previously described, mostly in the vagina, as a protective therapeutic option against candidiasis^24^. A key role of hydrogen peroxide and lactic acid production in this antifungal function is well documented. However, lactic acid was not detected in the metabolomic dataset. At the functional level, higher parasites carriage was associated with enrichment of metabolic activities related to replication and repair, translation and transcription. On the opposite, xenobiotics biodegradation and metabolism (amino acid metabolism, aminobenzoate and chlorocyclohexane/chlorobenzene degradation) were enriched in individuals harbouring low parasite diversity.

Finally, our results strengthen the observation made by Audebert et al.^8^ on a potentially positive association between parasitic colonization and microbial diversity and richness. The features observed in Ent3 individuals, at both compositional and functional levels, emphasize potential health complication notably gut inflammation.

Eventhough one cannot presently decipher the causal link between these two types of microorganisms, presence of gut parasites may partly be responsible or associated with the specific microbiome described in this population. While Pasolli et al. suspected a role of the nutrition to explain the very low proportion of *Bacteroides*, toward increased proportions of *Proteobacteria*, mostly *Enterobacteriaceae* and *Succinivibrionaceae* (previously undescribed bacterial genus *Succinatimonas*)^16^, we demonstrate that parasitism is a very strong driver of the human gut microbiome composition, diversity and functionality, which overtake the impact of diet.

## Conclusion

On the overall, our study highlights the existence of three specific enterotypes in the Malagasy population. Whereas diet, as previously described in other reports, is one of the most important drivers of the gut microbiota composition, individual protozoans carriage (understudied in westernized countries) accounts for most of the dissimilarity observed in the microbiome of people living in Madagascar. Further studies are needed to understand whether gut microbial richness constitute a predilection niche for protozoans colonization, probably due to their gazing features, or whether the parasites themselves induce a higher bacterial richness. Animal studies may help answering this question.

## Materials and methods

### Study population and design

The study was conducted prospectively from February to June 2015 in Mahajanga, North-West Madagascar, and its rural neighbourhood^14^. The region is characterized by tropical climate. Fecal samples from 219 Malagasy individuals, either healthy volunteers or patients hospitalized in the gastroenterology department of the university hospital PZaGa of Mahajanga, were collected. Two grams of feces were aliquoted and stored at − 20 °C before being routed to France in dry ice. Physicians concomitantly documented individuals’ socioeconomic, demographic and medical information, as well as dietary habits from each individual (Table 1). Exclusion criteria included the use of antibiotic or probiotic 2 months prior to inclusion and/or an age under 18 year-old.

**Table 1:** Characteristics of the 219 Malagasy individuals.

	Total	Parasites load (%)					
		0	1	2	3	4	> 4
n	219	33	73	44	24	21	24
**Parasitology**							
*Blastocystis*	77.63	0	86.3	90.91	100	90.48	100
*Dientamoeba*	22.83	0	1.37	34.09	20.83	66.67	62.5
*Giardia*	9.59	0	4.11	9.09	16.67	19.05	25
*E. hartmanni*	9.13	0	1.37	0	20.83	23.81	37.5
*E. nana*	10.05	0	1.37	2.27	20.83	23.81	41.67
*E. coli*	30.59	0	4.11	36.36	54.17	76.19	79.17
Amibe	3.65	0	0	2.27	16.67	9.52	4.17
*E. histolytica/dispar*	11.42	0	0	6.82	25	19.05	50
*C. mesnili*	10.96	0	0	2.27	12.5	23.81	62.5
*T. intestinalis*	2.74	0	0	0	0	9.52	16.67
*Embadomonas*	3.2	0	0	0	4.17	4.76	20.83
*Enteromonas*	8.68	0	0	4.55	4.17	28.57	41.67
*H. nana*	2.28	0	0	4.55	4.17	0	8.33
Ankylostomidae	3.2	0	1.37	6.82	0	4.76	8.33
**Demography**							
Age (mean)	–	37.21	34.48	31.02	30.17	27.48	28.42
Sex (M/F)	–	48.48	52.05	40.91	29.17	71.43	45.83
BMI (mean)	–	21.94	21.7	21.39	21.17	22.05	21.71
Geographic areas							
0	5.48	9.09	10.96	2.27	0	0	0
1	19.63	12.12	13.7	15.91	29.17	38.1	29.17
2	6.85	12.12	8.22	9.09	0	0	4.17
3	2.28	3.03	1.37	2.27	0	4.76	4.17
4	6.39	6.06	5.48	11.36	8.33	4.76	0
5	26.48	12.12	26.03	31.82	37.5	14.29	37.5
6	18.72	18.18	17.81	15.91	16.67	38.1	12.5
7	6.39	15.15	8.22	4.55	4.17	0	0
8	3.65	9.09	1.37	2.27	4.17	0	8.33
9	3.2	3.03	5.48	2.27	0	0	4.17
10	0.91	0	1.37	2.27	0	0	0
Sanitary level							
Low	78.08	63.64	76.71	72.73	87.5	90.48	91.67
Medium	15.53	30.3	17.81	15.91	4.17	4.76	8.33
High	6.39	6.06	5.48	11.36	8.33	4.76	0
**Diet**							
Liquid24h							
< 0.75	17.35	6.06	12.33	15.91	33.33	19.05	33.33
1	47.49	48.48	52.05	45.45	45.83	42.86	41.67
1.5	23.74	33.33	23.29	22.73	20.83	28.57	12.5
> 1.5	11.42	12.12	12.33	15.91	0	9.52	12.5
Fruit24h							
0	53.42	36.36	47.95	65.91	62.5	47.62	66.67
1	12.33	12.12	10.96	15.91	12.5	9.52	12.5
2	24.2	27.27	30.14	13.64	20.83	33.33	16.67
> 2	10.05	24.24	10.96	4.55	4.17	9.52	4.17
Rice24h							
< 300	17.35	21.21	12.33	18.18	29.17	4.76	25
400	25.57	18.18	21.92	13.64	41.67	33.33	45.83
500	29.22	24.24	34.25	38.64	16.67	33.33	12.5
> 500	27.85	36.36	31.51	29.55	12.5	28.57	16.67
Meat							
No	19.18	18.18	27.4	22.73	0	19.05	8.33
Yes	80.82	81.82	72.6	77.27	100	80.95	91.67
Yogourt HM							
No	49.77	39.39	52.05	52.27	45.83	47.62	58.33
Yes	50.23	60.61	47.95	47.73	54.17	52.38	41.67
Yogourt I							
No	89.04	84.85	87.67	84.09	100	95.24	91.67
Yes	10.96	15.15	12.33	15.91	0	4.76	8.33

Data are summarized according to protozoan carriage. Individuals' age and BMI are expressed as the mean value computed for each groups. Sex is expressed as the male to female ratio. Protozoan detection, dietary habits, sanitary levels and geographic areas are expressed as the percentage of individuals encountered in each group of parasites load. Information related to the geographic areas can be found in Greigert et al.^14^.

### Bacterial diversity and composition assessment by high-throughput sequencing

After a bead-beating step, total DNA was extracted from 200 mg of feces using the DNA Stool minikit (Qiagen) according to manufacturer recommendations. Bacterial load was assessed by quantitative PCR of the Bacteria domain as previously described^25^. The V3–V4 region of the 16S rRNA gene was amplified with the following primers: V3F «TACGGRAGGCAGCAG» (bac339F modified from^26^) and V4R «GGACTACCAGGGTATCTAAT» bac806R. 16S rDNA amplicon libraries were sequenced at GENOTOUL (https://www.genotoul.fr) on a MiSeq device using the 2 × 250 bp V3 kit. Remaining adapter/primer sequences were trimmed and reads were checked for quality (≥ 20) and length (≥ 200 bp) using cutadapt^27^. Reads were further corrected for known sequencing error using SPAdes^28^ and then merged using PEAR^29^. OTUs were identified using a Vsearch pipeline^30^ set up to dereplicate (–derep_prefix –minuquesize 2), cluster (–unoise3), chimera check (uchime3_denovo) the merged reads. OTU taxonomical classification was performed using both classifier and seqmatch from RDPTools suit^31^. The training sequence data set required for OTU classification at species level was downloaded from EzBioCloud 16S database for QIIME^32^. Metagenomic profile inference from 16S dataset was determined using PICRUSt^33^.

### Parasitological examination

All fecal samples were processed in the 30 min following stool emission using both culture (Dobell and Laidlaw biphasic medium) and culture-independent approaches as previously described^14^. Briefly, culture-independent assays relied on microscopy examinations of both non- and concentrated faeces (MIF and Faust methods). Molecular diagnosis was carried out for *Blastocystis, D. fragilis, Cryptosporidium, E. bieneusi* and *E. intestinalis*. We discriminated between *E. dispar* and *E. histolytica* by using conventional PCR. Detailed information, including primer sequences, can be found in Greigert et al.^14^. Stool samples were considered positive if helminth eggs, larvae, cysts and/or trophozoites of protozoans were detected by at least one of the four conventional methods and/or a positive molecular diagnosis.

### Microbial metabolomics

Eighteen fecal samples were selected for metabolomics analysis by Profilomic (http://www.profilomic.com/fr). Aliquots (50 mg) were suspended in 50 µL of water and 400 µL of methanol, centrifuged at 4 °C and 10.000*g* for 15 min and precipitated for 1 h 30 at 4 °C. Following second centrifugation and cleaning, supernatant was evaporated under N_2_ atmosphere. The pellet was suspended in 250 µL mix of water and AcN (40/60) containing internal standards. The solution was centrifuged at 20,000*g* and 4 °C for 15 min and injected in the mass spectrometer (Q-Exactive—ThermoFisher Scientific) coupled to liquid chromatography (aSequant ZIC-pHILIC—Merck) machine (LC-HRMS). The analysis was run at high resolution (70,000 FWHM) with alternating negative and positive ionisation mode. Signals were annotated using mass and retention time from each spectrum and a metabolite database issued from Profilomic.

### Statistics

Statistical analyses were run using R programming language and software together with gplots, gdata, vegan (http://cran.r-project.org/package=vegan), ade4 and phangorn^34^ packages. OTU counts were normalized via simple division to their sample size and then multiplication by the size of the smaller sample. α-Diversity and richness were estimated using *diversity* and *estimateR*. Distance matrix for β-diversity analysis was computed using *vegdist* and Bray–Curtis method. Principle Component Analysis and Principle Coordinate Analysis were computed either on compositional data or on distance matrix data using *dudi*.*pca* and *dudi*.*pco* respectively. Effects of demography, diet, presence of parasite on β-diversity were assessed using *adonis* function together with Bray–Curtis distance matrix. Wilcoxon test, Kruskal Willis test and anova were used as required to detect differences between groups of variables. P-values were corrected as necessary using False Discovery Rate correction. Enterotypes were defined as previously published^15^.

### Ethics approval and consent to participate

This study was approved by the Malagasy Ministry of Public Health (reference number 33-MSANP/CE). Written informed consents were obtained from all participants. This study was conducted in accordance with the Code of Ethics of the World Medical Association (Declaration of Helsinki).

## Supplementary Information

Supplementary Legends.

Supplementary Figure S1.

Supplementary Figure S2.

Supplementary Table S1.

Supplementary Table S2.

Supplementary Table S3.

Supplementary Table S4.

Supplementary Table S5.

## Data Availability

Data are available from the corresponding author. 16S rRNA gene reads are publicly available from NCBI SRA under Bioproject accession number PRJNA600229.
