# Supplementary material for: Parasites and diet as main drivers of the Malagasy gut microbiome richness and function
Source: Sci Rep. 2021 Sep 3;11:17630. doi: 10.1038/s41598-021-96967-4 (PMC8417078; doi:10.1038/s41598-021-96967-4)
Supplement: Supplementary file 1 — Supplementary Legends. [file 41598_2021_96967_MOESM1_ESM.docx]

## Additional information

## Additional file 1: Figure S1 (.tif)

1. Bacterial diversity (Simpson Index) and richness (OTU number) displayed according to enterotypes classification.
2. Bacterial load according to enterotypes classification. The bacterial load is expressed as log_10_ of CFU per gram of faecal content.

## Additional file 2: Figure S2 (.tif)

1. Bacterial richness (OTU number), load and diversity (Simpson Index) according to the presence/absence of mentioned protozoans.
2. Proportion of individuals living in area with different sanitary levels (as described in [[14](#_ENREF_14)], from low sanitary level (dark grey) to high standard sanitary levels (light grey), in relation to cumulative number of different protozoans.
3. Proportion of individuals included in each enterotype according to specific protozoan.
4. Principal coordinates analysis based on OTU composition displaying individuals coloured according to the cumulative number of Protists, from none (blue) to more than 4 (red). Individuals clustering within enterotypes are described by coloured ellipses (blue for *Ruminococcus*-driven Ent1, green for *Clostridium*-driven Ent2 and red for *Escherichia*-driven Ent3).
5. Relative abundance of *Acetanaerobacterium* and *Flavonifractor* according to the detection of *Blastocystis* (+ and -) and gastrointestinal symptoms (A: Asymptomatic and S: Symptomatic).
