# Supplementary figures and images for: Parasites and diet as main drivers of the Malagasy gut microbiome richness and function
Source: Sci Rep. 2021 Sep 3;11:17630. doi: 10.1038/s41598-021-96967-4 (PMC8417078; doi:10.1038/s41598-021-96967-4)

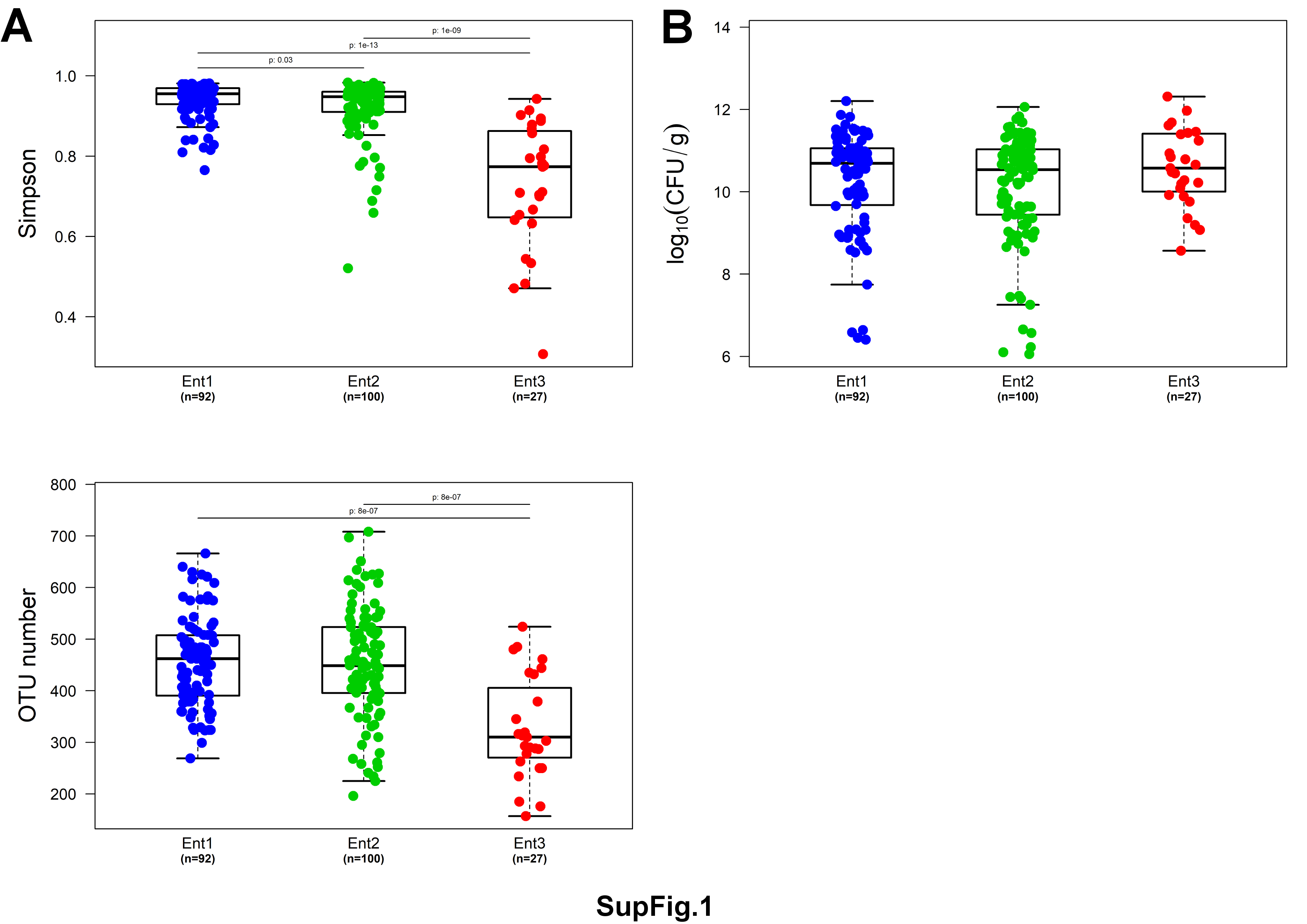

Supplement: Supplementary file 2 — Supplementary Figure S1. [file 41598_2021_96967_MOESM2_ESM.tif]

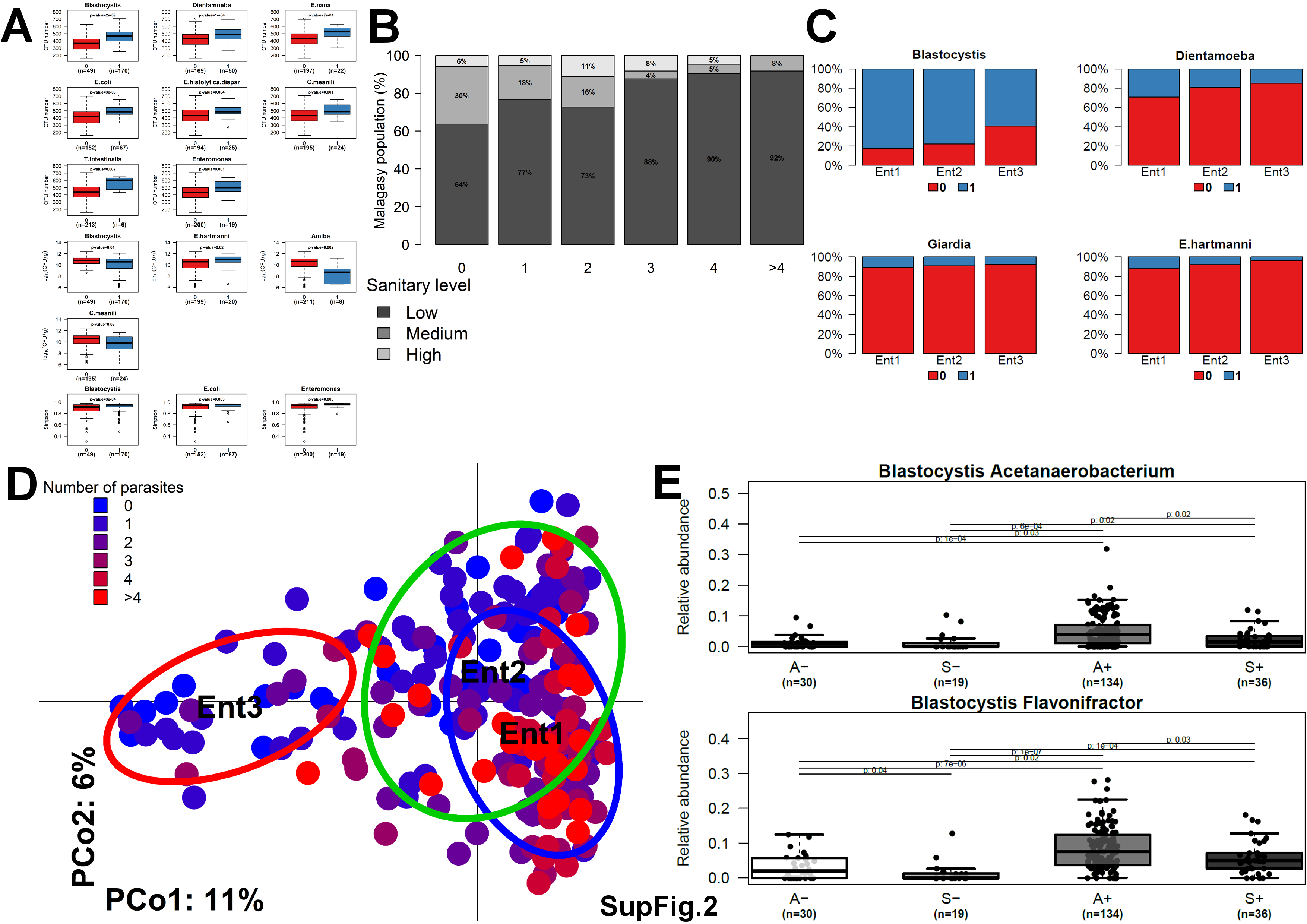

Supplement: Supplementary file 3 — Supplementary Figure S2. [file 41598_2021_96967_MOESM3_ESM.tif]
